# Corosolic Acid Inhibits Hepatocellular Carcinoma Cell Migration by Targeting the VEGFR2/Src/FAK Pathway

**DOI:** 10.1371/journal.pone.0126725

**Published:** 2015-05-15

**Authors:** Chung-Yu Ku, Ying-Ren Wang, Hsuan-Yuan Lin, Shao-Chun Lu, Jung-Yaw Lin

**Affiliations:** 1 Institute of Biochemistry and Molecular Biology, College of Medicine, National Taiwan University, Taipei, 100, Taiwan; 2 Department of Life Science, National Taiwan Normal University, Taipei, 116, Taiwan; Taipei Medicine University, TAIWAN

## Abstract

Inhibition of VEGFR2 activity has been proposed as an important strategy for the clinical treatment of hepatocellular carcinoma (HCC). In this study, we identified corosolic acid (CA), which exists in the root of *Actinidia chinensis*, as having a significant anti-cancer effect on HCC cells. We found that CA inhibits VEGFR2 kinase activity by directly interacting with the ATP binding pocket. CA down-regulates the VEGFR2/Src/FAK/cdc42 axis, subsequently decreasing F-actin formation and migratory activity *in vitro*. In an *in vivo* model, CA exhibited an effective dose (5 mg/kg/day) on tumor growth. We further demonstrate that CA has a synergistic effect with sorafenib within a wide range of concentrations. In conclusion, this research elucidates the effects and molecular mechanism for CA on HCC cells and suggests that CA could be a therapeutic or adjuvant strategy for patients with aggressive HCC.

## Introduction

Hepatocellular carcinoma (HCC) is the fifth most commonly occurring cancer and the third most common cause of death from cancer [1]. Although the prognosis of HCC is poor, surgical resection and liver transplantation often have curative effects in patients [2]. Previous studies have implicated emerging pathways in HCC, such as HGF/MET, Wnt/β-catenin, and VEGF/VEGFR; these pathways could serve as novel molecular targets for developing anti-HCC therapies [3–5].

Cancer cell migration is a critical process in tumor development and metastasis [6, 7], and thus, anti-migration therapy is considered to be an important approach for HCC treatment. VEGF receptor signaling, specifically VEGFR2 (KDR), has been implicated in HCC migration. Phosphorylated VEGFR2 can induce downstream kinase activation, such as Src, FAK, and Rho-GTPase, resulting in remodeling of actin filaments and induction of migratory activity of tumor cells [8–10]. Previous studies have shown that VEGFR knockdown reduces HCC cell migration [11]. Small molecule inhibition of VEGFR2 also reduces cancer cell migration [12, 13] and tumor growth *in vivo* [14–16].

Chinese herbal medicines (CHMs) have been used as potential therapies for a variety of human diseases, including hypertension, inflammation, and cancer [17]. Recent studies suggest that CHMs could be used to improve the efficiency of conventional cancer therapies and relieve side effects of chemotherapies [18]. In this study, we showed that corosolic acid (CA), which was found in the water extracts of Actinidia chinensis, exhibited significant anti-cancer effects in HCC cells by decreasing HCC cell migration without cytotoxicity. We further found that CA significantly inhibited VEGFR2 kinase activity by blocking the ATP binding site, resulting in down-regulation of the VEGFR2/Src/FAK/cdc42 signaling pathway. In vitro and in vivo synergistic analyses suggested that CA could be a potential agent for anti-HCC therapy.

## Materials and Methods

### Plant extracts

Water extracts from *A*. *chinensis* were supplied by the Sun Ten Pharmaceutical Company (Taipei, Taiwan). The plant materials were boiled in water and concentrated to 1 g/ml with an evaporator, and the stock solutions were stored at −20°C until use.

### HPLC analysis

We analyzed the constituent distribution and content in the water extracts of *A*. *chinensis* by high-performance liquid chromatography-diode array (HPLC-DAD)/evaporative light scattering detector (ELSD) chromatography under the following conditions: a linear gradient of ddH_2_O to methanol for 60 minutes, and 100% methanol for another 10 minutes at a flow rate of 1mL/minute with DAD/ELSD.

### Reagents

Corosolic acid, ursolic acid, 3-(4,5-Dimethylthiazol-2-yl)-2,5-diphenyltetrazolium bromide (MTT), and sulphorhodamine (SRB) were obtained from Sigma-Aldrich. Sorafenib was purchased from Santa Cruz Biotechnology. Lipofectamine 2000, VEGFR2 (KDR) siRNA, phalloidin, and Alexa Flour Dyes were obtained from Invitrogen Life Technologies. The primary antibodies against VEGFR2, p-VEGFR2 (Tyr1054), p-VEGFR2 (Tyr951), Src, p-Src (Tyr416), FAK and p-FAK (Tyr397) were purchased from Cell Signaling Technology. The Matrigel Matrix was obtained from BD Biosciences.

### Cell culture

The HCC cell lines: Huh7, HepG2 and Hep3B were obtained from Japanese Collection of Research Bioresources (National Institute of Health Sciences; Japan, JCRB) and maintained in Dulbecco’s Modified Eagle Medium-High Glucose (Invitrogen) medium with 10% fetal bovine serum (FBS), 2mM L-glutamine (Invitrogen), and 100 μg/mL penicillin-streptomycin (Invitrogen). Cells were cultured in a humidified atmosphere in 5% CO_2_ at 37°C.

### Cytotoxicity assay

To study the cytotoxicity of CA, the MTT assay was performed as described previously [19]. Huh7 cells were seeded at 5 × 10^3^ cells/well in 96-well plates and treated with 0.1% DMSO (control) or various concentrations of CA for 24 h. The number of viable cells was estimated by measuring the conversion of tetrazolium salt MTT to formazan crystals. After incubation with MTT for 6 h, the formazan crystals were solubilized with an SDS solution (10% SDS and 0.01M HCl) and quantified by measuring the absorbance at 590 nm with a reference wavelength of 650 nm.

### Migration assay

In the upper chamber, Huh7 cells (5 × 10^4^ cells) were starved overnight and resuspended in 300 μL serum-free DMEM medium with 0.1% DMSO (control) or various concentrations of CA and seeded into Transwell inserts (8 μm pore; BD Biosciences). The complete DMEM medium was added to the lower chamber, and then incubated for 16 h; the migrated cells were fixed, stained with crystal violet, and quantified in 3 random fields (40x magnification) per insert [19].

### Immunoprecipitation

For immunoprecipitation, Huh7 cells were treated with 0.1% DMSO (control) or CA for 15 min and lysed in RIPA buffer. The lysates were then sonicated and centrifuged, and the supernatant was incubated with anti-VEGFR1, R2, and R3 antibody (Santa Cruz Biotechnology, Inc.) overnight at 4°C. The immune-complexes were then incubated with PureProteome magnetic beads (Millipore) for 1 h at 4°C, washed and eluted with protein sample buffer, and analyzed by western blotting.

### Western blot analysis

Cells were collected at the indicated time points and protein was extracted with RIPA buffer. Proteins samples were analyzed by SDS-PAGE, transferred to PVDF membrane, and blocked with 5% milk in TBST. Membranes were then incubated with the following primary antibodies against VEGFR2, p-VEGFR2 (Tyr1054), p-VEGFR2 (Tyr951), Src, p-Src (Tyr416), FAK and p-FAK (Tyr397). After incubation with an HRP-conjugated secondary antibody (Santa Cruz Biotechnology) membranes were developed with ECL reagent (Millipore). Signals were captured with an LAS-3000 image capture system (Fuji) and quantified with ImageJ software [20].

### Kinase activity assay

The experiment was performed with the ADP-Glo kinase assay kit (Promega, WI, USA). Briefly, CA was first diluted with kinase reaction buffer at a 1:2 dilution ratio in different tubes (starting from 1 mM). Three nanograms of KDR (#V2681, Promega) were added to each tube and incubated for 10 min. Then, 0.1 μg/μL substrate and 10 μM ATP were added to each tube and incubated for 1h at room temperature. Next, 25 μL ADP-Glo reagent was added to the mixture and incubated at room temperature for 40 min. Finally, 50 μL kinase detection reagent was added to introduce luciferase and samples were measured with a SpectraMax L Microplate reader (Molecular Device, CA, USA).

### Rho GTPase activity assay

Huh7 cells were treated with 0.1% DMSO (control) or CA for 6 h and collected in RIPA buffer. Whole cell lysates (500 μg) were combined with purified GST fusion protein conjugated with Rac1, RhoA, or cdc42 binding domain (PAK-PBD for Rac1 and cdc42, Raf-RBD for RhoA) and incubated with head-to-head rotation at 4°C overnight [21]. MagneGST beads (Promega, WI, USA) were then added to the mixture to pull down the immune-complex. Samples were centrifuged at 14,000 rpm for 30 min, washed with RIPA buffer 5 times, boiled with SDS sample buffer, and analyzed by western blot analysis.

### G-actin/F-actin activity assay

The assay was performed as previously described [22]. To summarize, Huh7 cells were treated with 0.1% DMSO (control) or CA for 6 h and incubated in stabilizing buffer (1% Triton X-100, 1 μg phalloidin, and protease inhibitor cocktail) at room temperature for 5 min. Cell lysates were collected, followed by centrifugation at 100,000 ×g for 1 h at 37°C. The supernatant was removed and saved as the G-actin fraction. The pellet was washed twice with PBS and dissolved in 200 μL dissolving buffer (1% Triton X-100, 2% SDS, and protease inhibitor cocktail) by sonication twice, put on ice for 1 h, and saved as the F-actin fraction. Both fractions were then analyzed by western blotting.

### Confocal microscopy analysis

Huh7 cells were seeded on a 22 × 22 cover slide and treated with 0.1% DMSO (control) or CA for 6 h. At the indicated time, the cells were washed, fixed, and permeabilized with 0.25% Triton X-100 for 10 min. For double staining, the slides were first incubated with p-FAK (Tyr397) primary antibody overnight, and then stained with Alexa488 (anti-rabbit) and Alexa568-phallodin (20 mU/mL) for 1 h in darkness [23]. Finally, the samples were counter-stained for nuclei with DAPI (10 ng/mL) for 10 min. The images were captured and analyzed using the Leica TCS SP5 Spectral Confocal System. The actin filament intensity was measured by ImageJ (NIH) and calculated by the following formula [24]:

Correctedtotalcellfluorescence(CTCF)=IntegratedDensity–(Areaofselectedcell×Meanfluorescenceofbackgroundreadings)

### Animal model

All animal experiments were conducted according to the guidelines approved by the Institutional Animal Care and Use Committee of the College of Medicine, National Taiwan University, and the study were approved by the Animal Care and Use Committee at National Taiwan University. The male NOD/SCID mice (4–6 weeks old) were obtained from BioLASCO Taiwan Co., Ltd, and kept by Laboratory Animal Center of the College of Medicine, National Taiwan University. The experimental mice were housed into individually-ventilated cages (IVC), and free accessed to food and drinking water. For studying the anti-tumor effect of CA alone, Huh7 cells (2 × 10^6^ cells) were suspended in 200 μL of Opti-MEM (Invitrogen) and injected subcutaneously into the flanks of each mouse. After one week, the mice were treated with 50 μL DMSO (control) or CA (5 mg/kg/day) by intraperitoneal injection (n = 5 for each group) for 21 days. To study the combinatorial effect of CA and sorafenib, Huh7 cells (5 × 10^6^ cells) were suspended in 100 μL of Opti-MEM with matrigel-matrix (1:1 mix ratio), and injected subcutaneously into the flanks of each mouse. After one week, the mice were treated with 50 μL DMSO (control) and compounds by intraperitoneal injection (n = 5 for each group) for 20 days. The tumor volume was calculated by the following formula: tumor volume [mm^3^] = (length [mm]) × (width [mm])^2^ × 0.5. At the end of the experiment, the mice were anesthetized by Zoletil 50 (Virbac Animal Health) and sacrificed by CO_2_ euthanasia. The tumors were excised, weighed, and fixed for further studies.

### Immunohistochemistry

Samples for these experiments were obtained from the xenograft experiment and fixed in 10% paraformaldehyde, embedded in paraffin, and sectioned. The tissue sections were then subjected to immunohistochemical staining with the Novolink Polymer Detection System (Leica Biosystems). The sections were stained for p-VEGFR2 (Tyr951, Cell Signaling Technology), Ki-67 and p-FAK (Tyr397) (Santa Cruz Biotechnology), and the nuclei were counterstained with hematoxylin.

### Synergistic analysis

The synergistic analysis was analyzed by the Compusyn software, which was developed by Chou and Martin [25]. The software was used to estimate the combination index (CI) and *fa* (fraction affected by drugs) to study the combined effect of drugs. A CI < 1, CI = 1, and CI > 1 indicates synergistic, additive, and antagonistic effects, respectively.

### Molecular docking

The interaction of CA and the ATP-binding site in VEGFR2 was studied by Discovery Studio Modeling 4.0 and displayed by PyMOL (ver. 1.6.0b1). The structure of CA was obtained from ZINC (code: 08829484), and the crystal structure of VEGFR2 was obtained from Protein Data Bank (PDB id: 1YWN).

### SRB cell growth assay

Huh7, HepG2, and Hep3B cells were seeded into 96-well plates (5 × 10^3^ cells/well) and treated with 0.1% DMSO (control) or various concentrations of CA and sorafenib. After 24 hours, cells were fixed with 10% TCA and stained with SRB at 0.4% (w/v) in 1% acetic acid. The cells were then washed by 1% acetic acid, solubilized with 10 mM Tris base solution, and measured the absorbance by ELISA reader (515 nm wavelength).

### Statistical analysis

Data were presented as means with standard errors (SE) and analyzed with Prism 6 (GraphPad Software, Inc.) and Sigmaplot version 10 (Systat Software Inc.). One-way ANOVA was used to compare results with more than one treatment, and the Student’s *t*-test was performed to compare differences between two groups. *P* < 0.05 was considered statistically significant.

## Results

### Corosolic acid significantly decreases the migration activity of Huh7 cells

**Fig 1:**
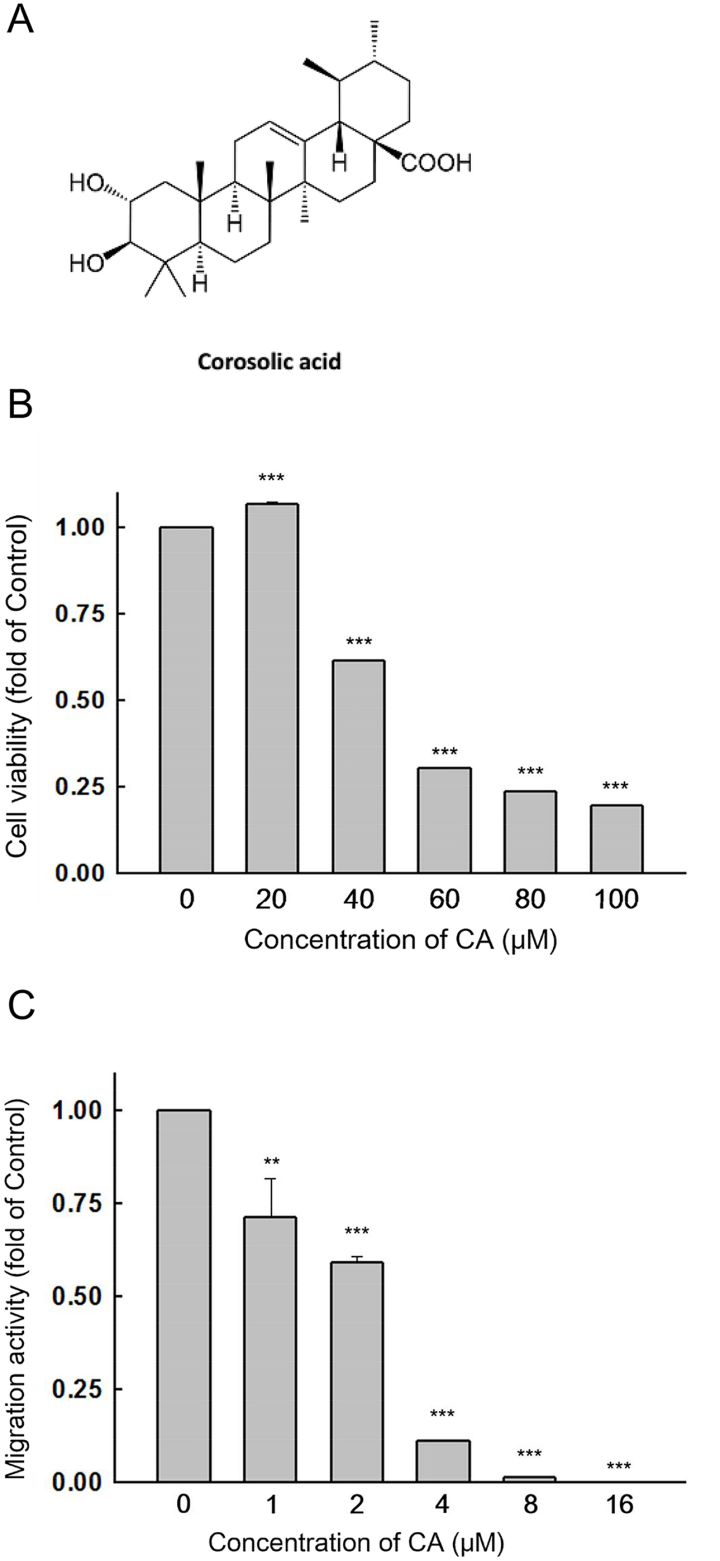
Migration activity of Huh7 cells is inhibited by corosolic acid without cytotoxicity. (A) Chemical structure of corosolic acid (B) Huh7 cells were treated with 0.1% DMSO (control) or various concentrations of corosolic acid for 24 h and cell viability was determined with an MTT assay. (C) The migration activity of Huh7 cells was inhibited by corosolic acid in a dose-dependent manner. (n = 3, **P < 0.01, ***P < 0.001 compared with the DMSO treated group)

To study anti-migration effects of corosolic acid (CA) on Huh7 cells *in vitro*, we first treated Huh7 cells with various concentrations of CA for 24 h. Cell viability was then measured with an MTT assay. As shown in Fig 1B, CA decreased the survival rate of Huh7 cells; the IC_50_ of cytotoxicity was determined to be 50 μM. Then, we performed a transwell assay with Huh7 cells. CA inhibited Huh7 cell migration in a dose-dependent manner and the IC_50_ for migration was found to be 2.5 μM (Fig 1C). The results indicate that CA has a relatively higher inhibitory effect on Huh7 cell migration than cell viability. (IC_50 cytotoxicity_/ IC_50 migration_ = 20).

### Corosolic acid inhibits VEGFR2 kinase activity

**Fig 2:**
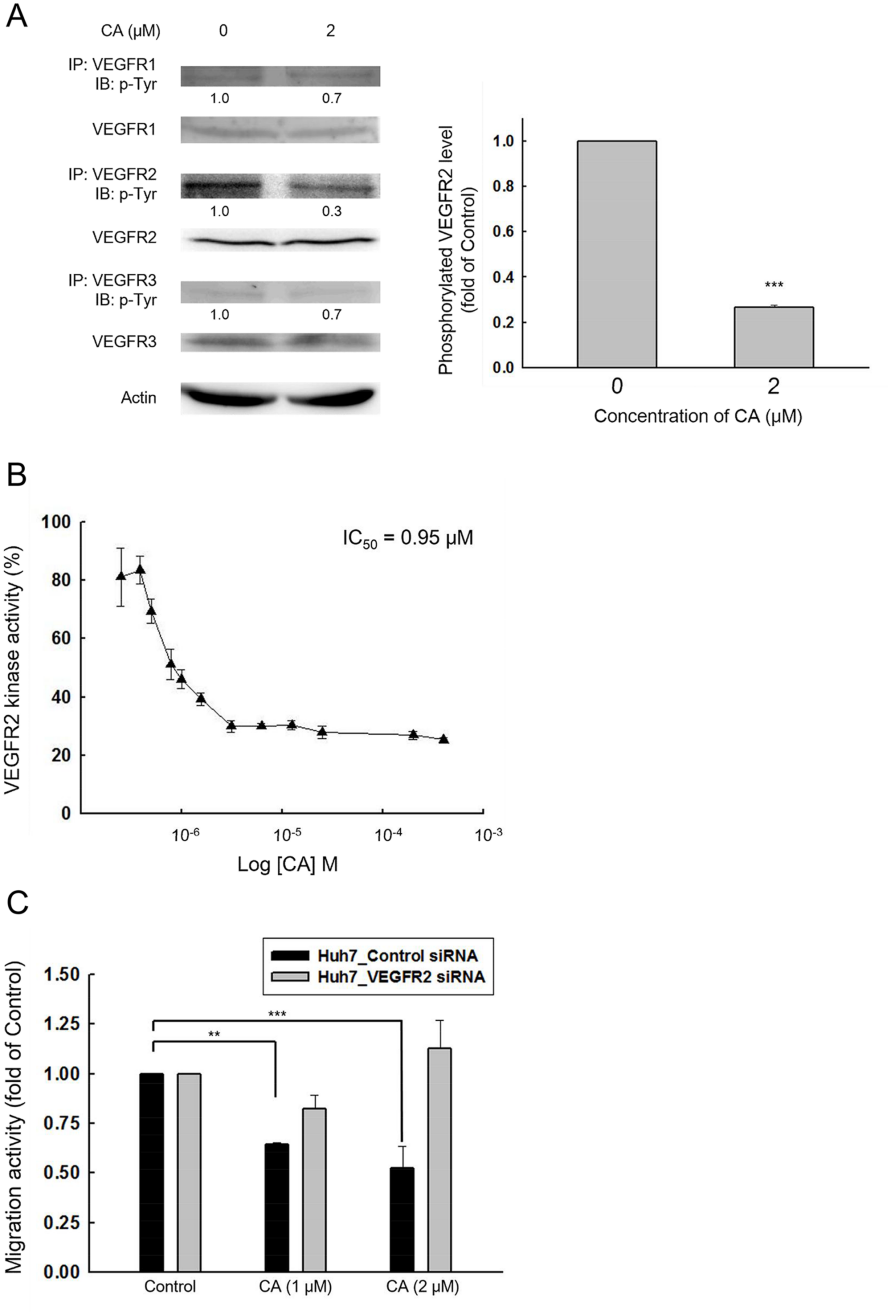
Corosolic acid reduces VEGFR2 kinase activity. (A) Huh7 cells were treated with 0.1% DMSO (control) or corosolic acid for 15 min and lysates were immunoprecipitated with anti-VEGFR1, VEGFR2, and VEGFR3 Ab, followed by blotting with anti-phospho-tyrosine Ab. (For VEGFR2, n = 3, ***P < 0.001 compared with the DMSO treated group) (B) ADP-Glo Kinase Assay (Promega, Madison, USA) was performed to assess the inhibitory effect of corosolic acid on VEGFR2 kinase activity. (n = 3, RLU data were normalized to the control group and shown as percentages) (C) Huh7 cells were transfected with 100 nM KDR siRNA or control siRNA, recovered for 24 h, and treated with 0.1% DMSO (control) or CA. Migration activity was assessed with a transwell assay. (n = 3, **P < 0.01, ***P < 0.001 compared with the DMSO treated cells in Huh7 control siRNA group)

Previous studies suggest that VEGF/VEGFR signaling can facilitate cancer cell metastasis [7], and inhibition of VEGFR can reduce HCC cell migration [11]. Thus, to investigate whether CA inhibits VEGFR activation, we performed immunoprecipitation to pull down three key VEGFRs in Huh7 cells, including VEGFR1, R2, and R3, followed by blotting with phospho-tyrosine antibody. The results suggest that CA significantly reduced phosphorylation of VEGFR2 by 70% without affecting total VEGFR2 expression (Fig 2A). With a VEGFR2 kinase activity assay, 0.95 μM CA was also found to inhibit VEGFR2 activity by 50% (Fig 2B). To examine whether the anti-migration effect of CA is mediated by VEGFR2, we attenuated endogenous VEGFR2 of Huh7 cells by siRNA. The knockdown group lost sensitivity to CA-induced inhibition of migration (Fig 2C). Taken together, these data suggest that CA inhibits Huh7 cell migration by inhibiting VEGFR2 activation.

### Corosolic acid decreases cell motility by inhibiting VEGFR2/Src/FAK/cdc42 activity and actin rearrangement

**Fig 3:**
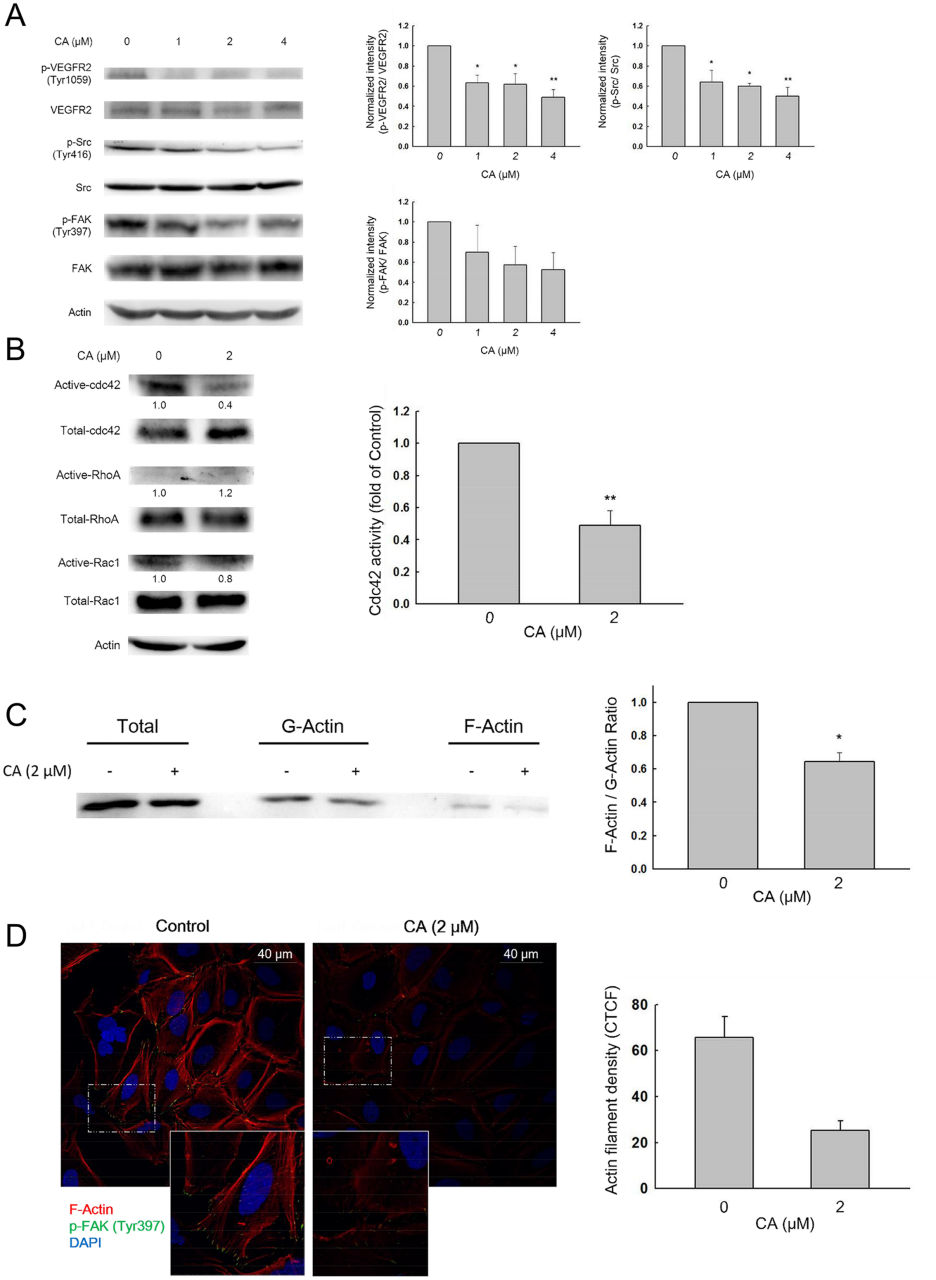
Effect of corosolic acid on VEGFR2-mediated downstream signaling and actin rearrangement. (A) Huh7 cells were treated with 0.1% DMSO (control) or corosolic acid for 30 min, and the phosphorylation level of VEGFR2 (Tyr1058), Src (Tyr416), and FAK (Tyr397) were analyzed by western blot. (n = 3, *P < 0.05, **P < 0.01 compared with the DMSO treated group) (B) Huh7 cells were treated with 0.1% DMSO (control) or corosolic acid for 6 h, and Rho-GTPase activity was examined with a GST pull-down assay and western blot analysis. (For cdc42, n = 3, **P < 0.01 compared with the DMSO treated group) (C) Huh7 cells were treated with 0.1% DMSO (control) or corosolic acid for 6 h, and fractions containing either F-actin or G-actin were separated by procedures outlined in materials and methods. The ratio of F-actin and G-actin were then calculated. (n = 3, ***P < 0.001 compared with the DMSO treated group) (D) Huh7 cells were treated with 0.1% DMSO (control) or corosolic acid for 6 h followed by immunocytochemistry staining. The phalloidin-stained F-actin (red) and p-FAK (green) co-localized at the leading edge of control cells.

To further elucidate the mechanism underlying the anti-migration effect of CA, we performed western blot analysis. Treatment with CA decreased the phosphorylation level of VEGFR2 (Tyr1058). The phosphorylation level of non-receptor tyrosine kinase, Src (Tyr416), and focal adhesion kinase, FAK (Tyr397), were also down-regulated by CA (Fig 3A). It was reported previously that focal adhesion kinase (FAK) is activated by membrane receptors and then the Src/FAK complex modulates cell migration and actin rearrangement via Rho-GTPase pathways. Therefore, using a Rho-GTPase activity assay, we found that active cdc42, but not Rac1 and RhoA, is significantly down-regulated by CA treatment (Fig 3B). Recent studies have revealed that cdc42 may play an important role in the dynamic change of actin and the formation of filopodia during cell migration. To study whether CA disrupts actin rearrangement in Huh7 cells, we performed a G-actin/ F-actin assay. The data demonstrated that CA treatment reduces the ratio of F-actin/G-actin (polymer/monomer) by about 50% compared to the control group (Fig 3C). By confocal microscopy analysis, we also found that CA decreases the co-localization of phospho-FAK (Tyr397) and F-actin on the filopodium (leading edge) in Huh7 cells (Fig 3D). Taken together, these data indicate that CA inhibits Huh7 cell migration by suppressing the VEGFR2/Src/FAK/cdc42 pathway and actin rearrangement.

### Corosolic acid exhibits anti-tumor effect *in vivo*

**Fig 4:**
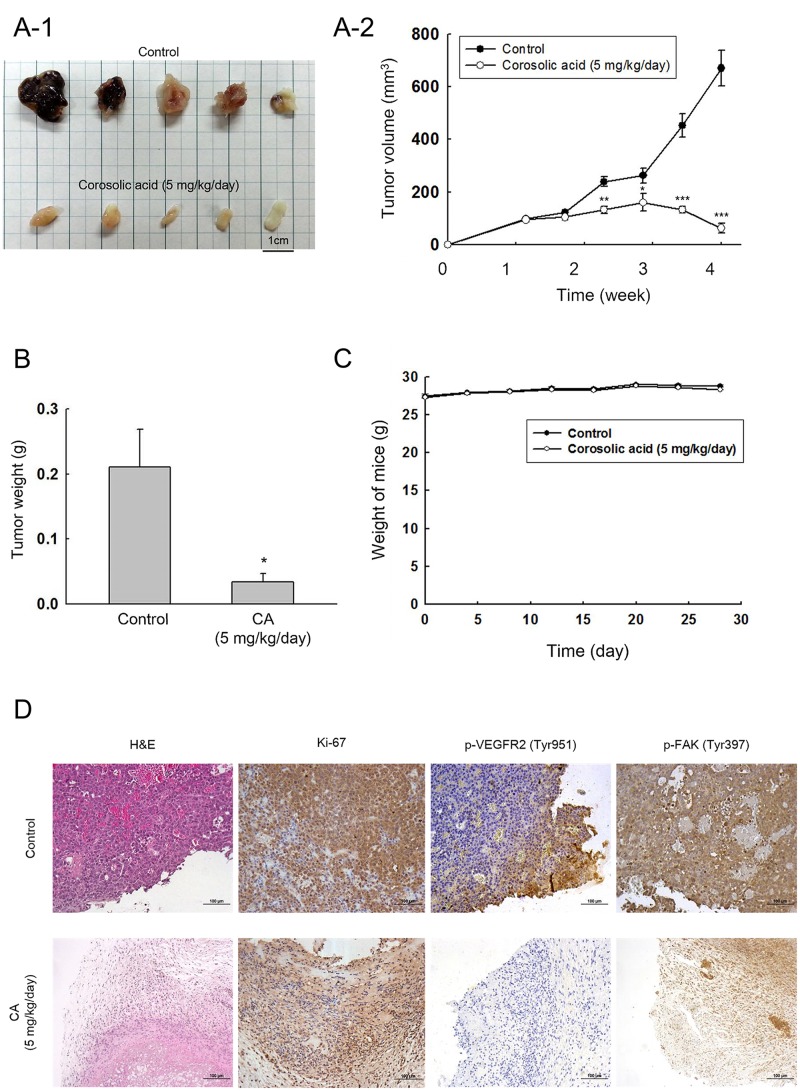
Corosolic acid exhibits significant anti-tumor effects on Huh7 cells *in vivo*. 2 × 10^6^ of Huh7 cells were subcutaneously injected into the hind limb of NOD/SCID mice (n = 5). Corosolic acid (5 mg/kg/day) was administered by intraperitoneal injection for 21 days. (A-1) Representative appearance of excised tumor. (A-2) Tumor volume, measured every 5 days. (*P < 0.05, **P < 0.01, ***P < 0.001 compared with the DMSO treated control group) (B) Weight of tumor mass. (*P < 0.05, compared with the DMSO treated control group) (C) Body weight between mice treated with and without corosolic acid. (D) Immunostaining of Ki-67, p-VEGFR2 (Tyr951) and p-FAK (Tyr397) in excised tumor in mice. Single staining was done on several sections.

The efficacy of CA on tumor growth were assessed *in vivo* using a xenograft model. Mice were given daily i.p. injection of CA (5 mg/kg/day). CA had significant inhibitory effects on tumor growth in NOD/SCID mice injected with Huh7 cells (2 × 10^6^ cells/mice) (Fig 4A). After 21 days of treatment, the mice were sacrificed and the volume of tumors in CA-treated group (63 ±19 mm^3^) were much smaller than that of control group (669 ±67 mm^3^). In addition, the CA-treated group (5 mg/kg/day) showed 85% reduction in tumor mass compared to the control group (Fig 4B). Body weight of mice treated with CA were similar to that of control group (Fig 4C), suggesting that the dosage of CA administered had little toxic effects to the mice. The levels of Ki-67, phospho-VEGFR2 and phospho-FAK in tumor lesions were examined by immunohistochemistry; CA reduced phosphorylation of both VEGFR2 and FAK significantly in HCC xenograft mice (Fig 4D).

### Synergistic effects of corosolic acid and sorafenib on HCC cells

**Fig 5:**
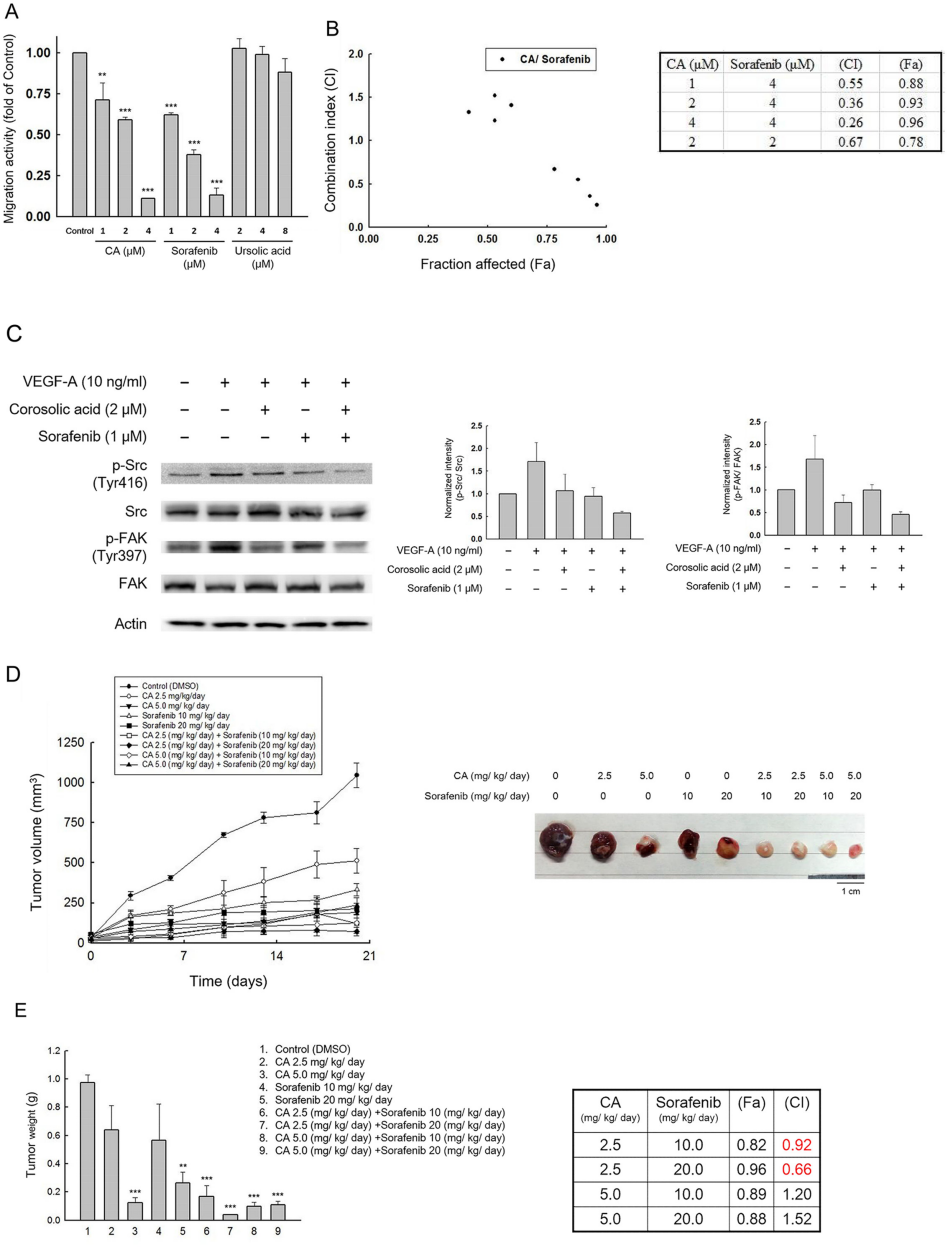
Combinatorial effects of corosolic acid and sorafenib on Huh7 cells. (A) Dose dependent migration inhibitory effects of corosolic acid and sorafenib on Huh7 cells. The control group was treated with 0.1% DMSO and the experimental groups were treated with indicated compounds. (n = 3, **P < 0.01, ***P < 0.001 compared with the DMSO treated group) (B) Transwell assay were performed to determine anti-migration effect of corosolic acid and sorafenib. The combination index (CI) values were examined at different levels of migration inhibition effect (fa), and the effective combination treatments between corosolic acid and sorafenib (CI < 1) were displayed. (C) Huh7 cells were treated with 0.1% DMSO (control), corosolic acid, sorafenib, or combination of corosolic acid and sorafenib for 30 min, and the lysates were analyzed by western blot. (D) For the *in vivo* combinatorial study, Huh7 cells (5 × 10^6^) were subcutaneously injected into each mouse. After 7 days, when the tumors reached 50 mm^3^, the mice were randomized into different groups. CA (2.5, 5 mg/kg), sorafenib (10, 20 mg/kg), or a combination of the two was administrated daily via intraperitoneal injection for 20 days the tumor volume was recorded every 3 to 4 days. (E) Weight of tumor mass (n = 5, **P < 0.01, ***P < 0.001 compared with the DMSO treated control group) and synergistic effects (CI < 1) between different combination group.

Sorafenib (Nexavar), a multi-kinase inhibitor including VEGFR2/3, PDGFRβ, and Flt-3, has been used to treat HCC patients and has a significant migration-inhibitory effect on HCC cells [26]. We performed a transwell assay with both CA and sorafenib treatment; CA exhibited a migration inhibitory effect comparable with sorafenib (Fig 5A). Ursolic acid (3β-hydroxyurs-12-ursen-28-ic acid) (UA) shares a similar chemical structure with CA and has been implicated in cancer prevention [27]. However, in the transwell assay, UA exhibited no significant anti-migration activity on Huh7 cells compared to CA. We demonstrated that CA has an inhibitory effect on migration comparable to sorafenib in HCC.

Next, to explore the effects of CA when used in combination with chemotherapeutic agents for HCC, we assessed the combinatorial effects of CA and sorafenib on migration activity of Huh7 cells. The transwell assay demonstrated that CA has a synergistic effect with sorafenib on cell migration at a wide range of doses (Fig 5B). To verify this, we performed a western blot analysis, and found that CA enhances sorafenib-mediated inhibition of phosphorylation of VEGFR2, Src, and FAK (Fig 5C). Finally, the xenograft model indicated that combined treatment with CA and sorafenib showed a synergistic effect on tumor growth (CA 2.5 mg/kg/day with sorafenib 10 or 20 mg/kg/day) (Fig 5D and 5E). These results demonstrate a synergistic interaction between CA and sorafenib in the treatment of HCC cells.

### Corosolic acid interacts with the ATP-binding site of VEGFR2 kinase domain by molecular docking

**Fig 6:**
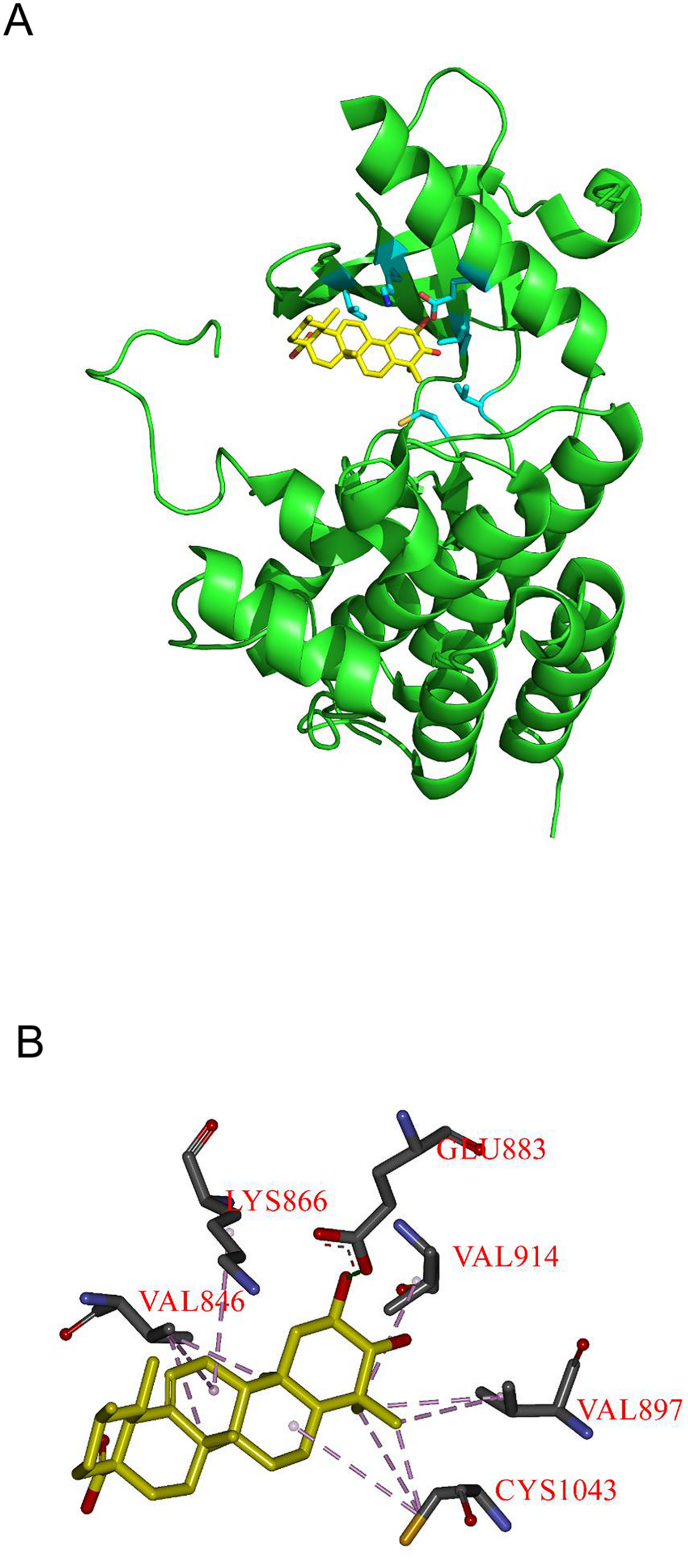
Corosolic acid interacted with the ATP-binding site of VEGFR2 kinase domain by molecular docking analysis. (A) The three-dimensional diagram displays the interaction of corosolic acid to the ATP-binding site of VEGFR2 (PDB code: 1YWN). (B) The interaction of corosolic acid with the amino acid residues in the ATP-binding site; Glu883 significantly contributes to binding.

To further study whether CA decreases phosphorylation of VEGFR2, we used molecular docking software to analyze the interaction between CA and the kinase domain of VEGFR2. This analysis suggests that CA may bind to the ATP-binding cavity of the VEGFR2 kinase domain (Fig 6A). Previous studies suggested that Gln883, Cys917, and Asp1044 of VEGFR2 are involved in ligand binding through H-bond interactions [28]. As shown in Fig 6B, CA potentially interacts with Gln883 at a distance of 2.67Å. It also interacts with Val846, Lys866, Val897, Val914, and Cys1043. These interactions between CA and the VEGFR2 kinase domain could result in inhibition of VEGFR2 and subsequent downstream intracellular signaling.

## Discussion

The present results indicate that corosolic acid (CA), displays anti-cancer activities *in vitro* and *in vivo* for the treatment of HCC cells. Specifically, CA exhibits anti-migratory activity, inhibits VEGFR2 kinase activity, disrupts actin filament formation, and has anti-tumor effects in a xenograft model. CA is a relatively potent inhibitor of VEGFR2 with IC_50_ values in the micromolar range. These results indicate that CA could be a potential chemotherapeutic agent for HCC therapy.

Anti-cancer effects of *A*. *chinensis* on cell proliferation, apoptosis, and angiogenesis have been noted in previous studies [29, 30]. We also observed that *A*. *chinensis* water extracts had an anti-cancer effect in Huh7 cells. *A*. *chinensis* was not cytotoxic to Huh7 cells between 0.5–4 mg/ml, and the IC_50 migration_ of *A*. *chinensis* was identified as 0.2 mg/ mL. (S1 and S2 Figs). Therefore, we performed HPLC analysis and identified the active component of *A*. *chinensis*, corosolic acid (CA), comprising about 8.4% of the dry weight of *A*. *chinensis* (S3 Fig).

CA is an ursane-type triterpenoid, and is known to be a STAT3 inhibitor in macrophages, myeloid cells, and ovarian cancer cells [31–33]. CA also has a significant inhibitory effect on endothelial angiogenic tube formation [30], and tumor growth in lung and ovarian cancer cells [32, 34]. In this study, we found that CA significantly reduced the migration activity of HCC cells, including Huh7 (Fig 1C), HepG2 (S4B Fig) and Hep3B (S5B Fig) at a low-cytotoxicity dosage. When combined with sorafenib, CA showed synergistic effects on HCC cell growth (S7 Fig) and migration (Fig 5B; S4C and S5C Figs). An *in vivo* xenograft mouse model was used to verify the anti-HCC activity of CA, which showed significant effects on Huh7 cells at 5 mg/kg/day (Fig 4).

VEGFR2 is the major receptor in the VEGF signaling pathway that regulates cell migration, proliferation, and angiogenesis. It is activated by VEGF binding, leading to trans/auto phosphorylation of intracellular tyrosine residues. The Tyr1054 and Tyr1059, located in the active loop of the kinase domain, are critical for VEGFR2 kinase activity; their phosphorylation induces downstream signal transduction, including Src and FAK [35, 36]. This study revealed that CA reduces the tyrosine phosphorylation level of VEGFR2, with an IC_50_ of kinase activity of 0.95 μM. Further studies also found that CA suppressed the activation of Src, FAK, and cdc42. These results provide a potential mechanism for the anti-migration effects of CA on Huh7 cells in HCC.

The inhibition of VEGFR2 has been proposed as a novel therapeutic strategy for HCC patients. Various VEGFR2 kinase inhibitors such as sorafenib, sunitinib, and linifanib were developed and used in clinical trials. Recently, anti-HCC therapy with sorafenib has been approved by FDA [26, 37]. To further investigate how CA inhibits VEGFR2, a structure-based interaction model between CA and VEGFR2 was developed by molecular docking analysis. The results suggest that the ATP binding pocket in the VEGFR2 catalytic domain binds CA with lower binding energy than ATP (-15.2 kcal/mol versus -12.3 kcal/mol). Moreover, the surface charge distribution of VEGFR2 demonstrated that the OH groups of CA showed stable interactions with the ATP binding pocket (S6 Fig). It also revealed that most uncharged areas of CA could generate hydrophobic forces with valine and cysteine resulting in stabilizing the binding affinity (Fig 6B). This strongly suggests that the binding of CA to the ATP-binding pocket of VEGFR2 mediates the down-regulation of VEGFR2 phosphorylation and subsequent signals. Furthermore, the combination of CA and sorafenib had significant synergistic effects on Huh7 cell migration and VEGFR2 phosphorylation (Fig 5B). The *in vivo* combinatorial experiment further verified that CA combined with sorafenib shows potential for HCC treatment without toxic effects to mice (data not shown). We also observed that CA down-regulated the phosphorylation level of Src and FAK kinases when combined with sorafenib, since sorafenib alone didn’t show any inhibitory effect to the activation of FAK kinase in the xenograft model (S8 Fig). Collectively, these results indicate that CA shows potential as a novel VEGFR2 inhibitor or an adjuvant therapy to be used with existing anti-cancer drugs.

Previous studies have discussed the pharmacophore modeling of different VEGFR2 inhibitors [28]. These inhibitors could be divided in two types, sunitinib-like or sorafenib-like, depending on the interacting hydrogen bonds. The binding of type I inhibitor (sunitinib) formed hydrogen bonds with Asp1044, Cys917, and Asn921 near the protein surface. On the other hand, the type II inhibitor (sorafenib) could interact with Asp1044, Cys917, and Glu883. By docking analysis, we found that CA formed hydrogen bond and relatively closed to Glu883 than Asn921 (2.67 Å versus 9.2 Å) (S9 Fig). Although the interaction model of CA with VEGFR2 are likely to sorafenib, however, the chemical structure of CA varied widely with both two types of VEGFR2 inhibitor. Thus, it could be interesting to explore and design novel VEGFR2 inhibitors based on our findings.

In conclusion, this study demonstrates that corosolic acid could be a potential anti-HCC agent. We provide evidence that CA’s anti-cancer effects stem from its anti-migratory effect, by blocking the VEGFR2 ATP binding pocket and down-regulating the downstream Src/FAK/cdc42 signaling axis. This study further demonstrates that the combination of CA and sorafenib may have potential as a chemotherapy for HCC.

## Supporting Information

S1 Fig Cytotoxicity of *Actinidia chinensis* on Huh7 cells. Huh7 cells were treated with 0.1% DMSO (control) or various concentrations of *A*. *chinensis* for 24 h and cell viability was determined with an MTT assay. Results are presented as mean value ± SE. (**P < 0.01, ***P < 0.001 compared with the DMSO treated group)
(PDF)

S2 Fig Migration activity of Huh7 cells was inhibited by *Actinidia chinensis*. The control cells were treated with 100 μL ddH_2_O, and the migration activity of Huh7 cells was inhibited by *A*. *chinensis* in a dose-dependent manner. Results are presented as mean value ± SE. (***P < 0.001 compared with the water treated group)
(PDF)

S3 Fig HPLC analysis of *Actinidia chinensis*. High-Performance liquid chromatography-diode array (HPLC-DAD)/ELSD chromatography was used to examine compounds in *A*. *chinensis*. The conditions for analysis are described in the methods section.
(PDF)

S4 Fig Cytotoxicity and migration-inhibitory effects of corosolic acid on HepG2 cells. (A) HepG2 cells were treated with 0.1% DMSO (control) or various concentrations of corosolic acid for 24 h and cell viability was determined with an MTT assay. Results are presented as mean value ± SE. (*P < 0.05, ***P < 0.001 compared with the DMSO treated group) (B) The migration activity of HepG2 cells was inhibited by corosolic acid in a dose-dependent manner. Results are presented as mean value ± SE. (***P < 0.001 compared with the DMSO treated group) (C) Combinatorial effects of corosolic acid and sorafenib on HepG2 cell migration are displayed by CI value.
(PDF)

S5 Fig Cytotoxicity and migration-inhibitory effects of corosolic acid on Hep3B cells. (A) Hep3B cells were treated with 0.1% DMSO (control) or various concentrations of corosolic acid for 24 h and cell viability was determined with an MTT assay. Results are presented as mean value ± SE. (***P < 0.001 compared with the DMSO treated group) (B) The migration activity of Hep3B cells was inhibited by corosolic acid in a dose-dependent manner. Results are presented as mean value ± SE. (**P < 0.01, ***P < 0.001 compared with the DMSO treated group) (C) Combinatorial effects of corosolic acid and sorafenib on Hep3B cell migration are displayed by CI value.
(PDF)

S6 Fig Surface charge distribution of VEGFR2 ATP binding pocket docking with corosolic acid. The surface charge distribution was displayed by PyMOL software. The negative charge, positive charge, and hydrophobic area were represented by red, blue, and white color, respectively.
(PDF)

S7 Fig Corosolic acid inhibits growth of Huh7, HepG2, and H3p3B cells. (A) Cells were treated with 0.1% DMSO (control) or varying concentrations of corosolic acid for 24 h, and the growth inhibition effect of corosolic acid was determined by SRB assay. Results are presented as mean value ± SE. (**P < 0.01, ***P < 0.001 compared with the DMSO treated group); combinatorial effects of corosolic acid and sorafenib on HCC cell growth are displayed on the right side of each chart.
(PDF)

S8 Fig Inhibitory effects of corosolic acid combined with sorafenib on Src and FAK kinases *in vivo*. Xenograft tumors excised from mice were homogenized in RIPA buffer and analyzed by western blotting. (n = 4 for each group, *P < 0.05, **P < 0.01 compared with the DMSO treated control group)
(PDF)

S9 Fig Relative distance between corosolic acid and interacting residues of VEGFR2. The Glu883 residue was represented by cyan color, and Asn921 residue was showed by pink color. The yellow dotted line means the distance between corosolic acid with these two residues.
(PDF)
