# Supplementary figures and images for: Corosolic Acid Inhibits Hepatocellular Carcinoma Cell Migration by Targeting the VEGFR2/Src/FAK Pathway
Source: PLoS One. 2015 May 15;10(5):e0126725. doi: 10.1371/journal.pone.0126725 (PMC4433267; doi:10.1371/journal.pone.0126725)

# S1 Figure

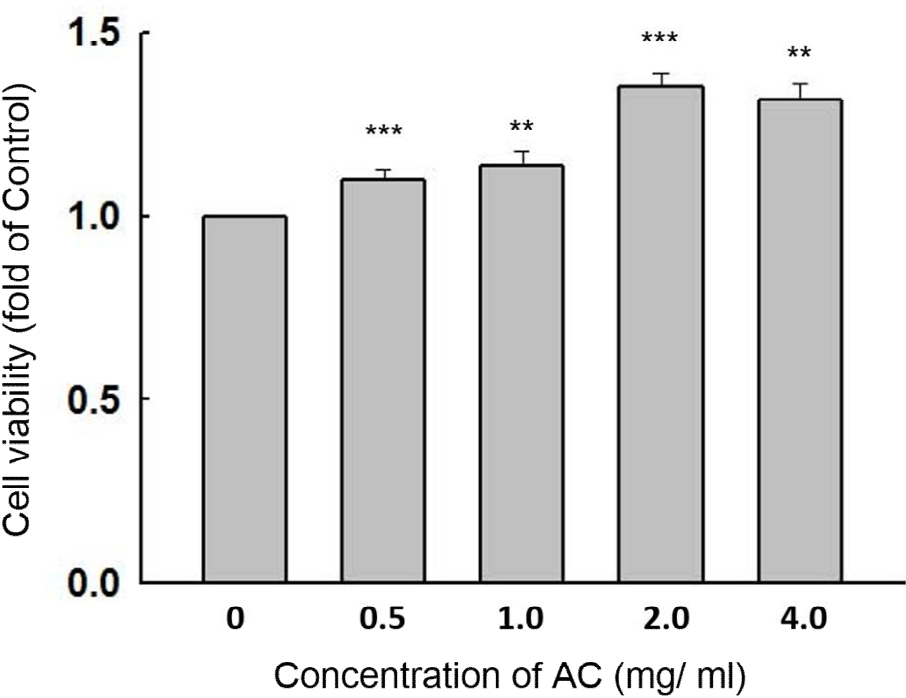

Supplement: S1 Fig — Huh7 cells were treated with 0.1% DMSO (control) or various concentrations of A. chinensis for 24 h and cell viability was determined with an MTT assay. Results are presented as mean value ± SE. (**P < 0.01, ***P < 0.001 compared with the DMSO treated group) (PDF) [file pone.0126725.s001.pdf]

# S2 Figure

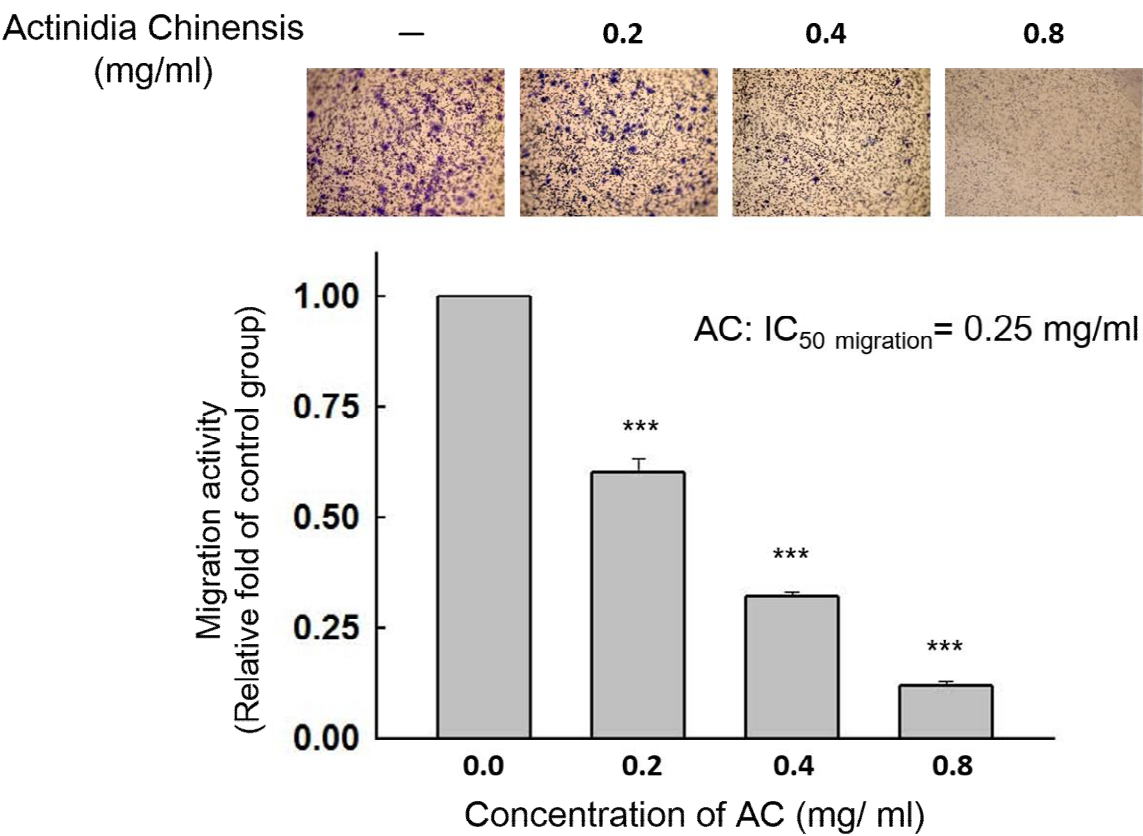

Supplement: S2 Fig — The control cells were treated with 100 μL ddH2O, and the migration activity of Huh7 cells was inhibited by A. chinensis in a dose-dependent manner. Results are presented as mean value ± SE. (***P < 0.001 compared with the water treated group) (PDF) [file pone.0126725.s002.pdf]

# S4 Figure

A

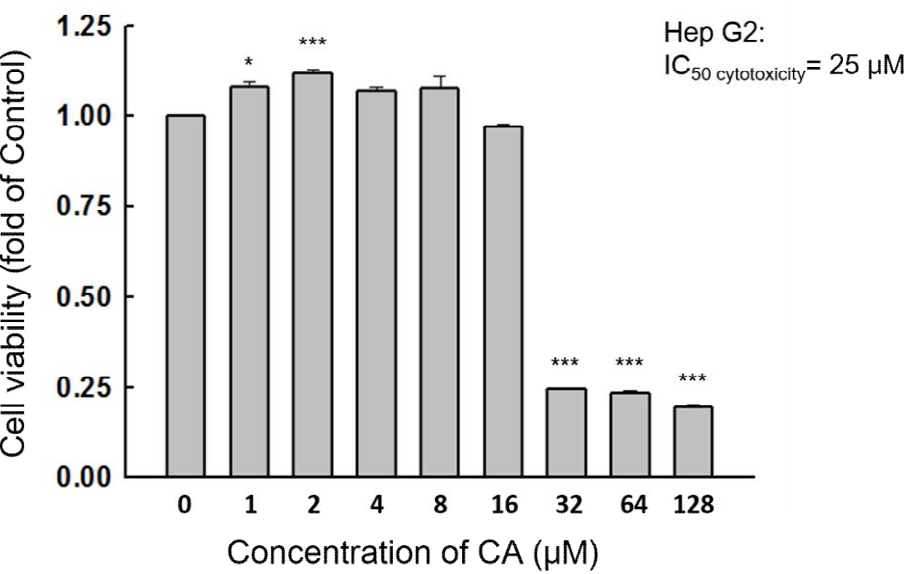

B

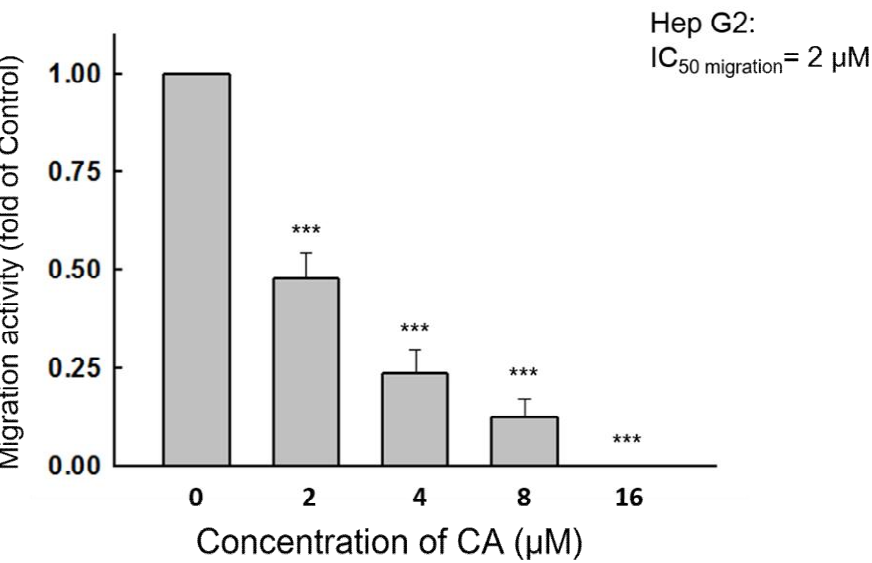

C

| HepG2          |         |      |      |
|----------------|---------|------|------|
| Sorafenib (µM) | CA (µM) | (Fa) | (CI) |
| 2.0            | 2.0     | 0.49 | 6.99 |
| 2.0            | 4.0     | 0.65 | 0.95 |
| 4.0            | 2.0     | 0.71 | 1.21 |
| 4.0            | 4.0     | 0.87 | 0.68 |

Supplement: S4 Fig — (A) HepG2 cells were treated with 0.1% DMSO (control) or various concentrations of corosolic acid for 24 h and cell viability was determined with an MTT assay. Results are presented as mean value ± SE. (*P < 0.05, ***P < 0.001 compared with the DMSO treated group) (B) The migration activity of HepG2 cells was inhibited by corosolic acid in a dose-dependent manner. Results are presented as mean value ± SE. (***P < 0.001 compared with the DMSO treated group) (C) Combinatorial effects of corosolic acid and sorafenib on HepG2 cell migration are displayed by CI value. (PDF) [file pone.0126725.s004.pdf]

# S5 Figure

A

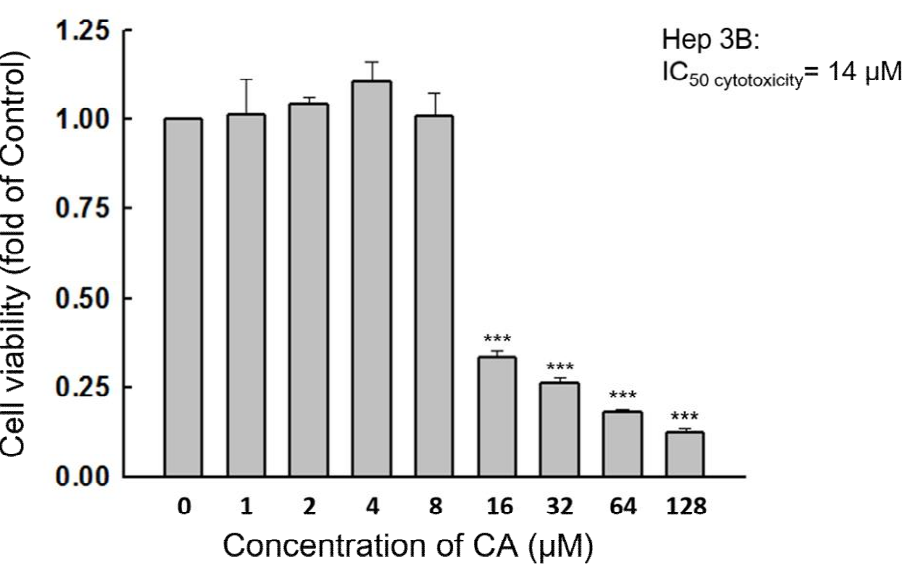

B

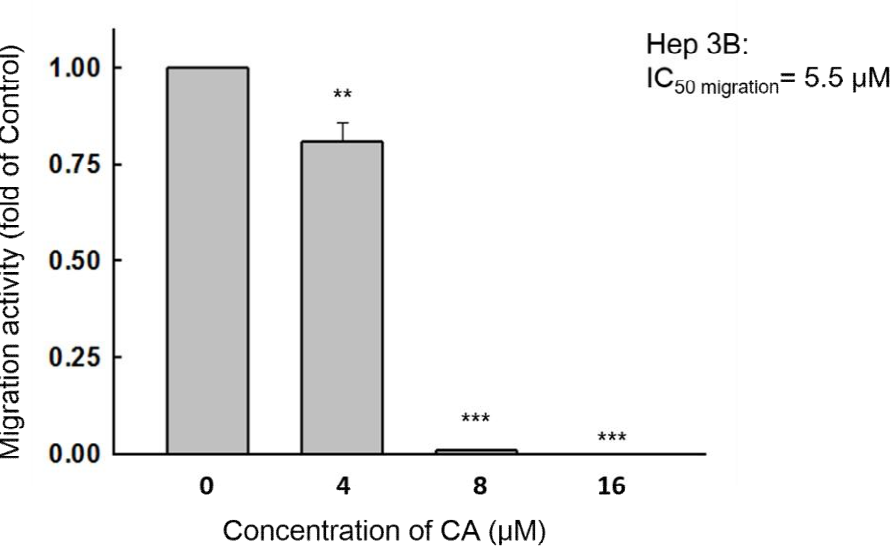

C

| Hep3B          |         |      |      |
|----------------|---------|------|------|
| Sorafenib (μM) | CA (μM) | (Fa) | (CI) |
| 5.0            | 5.0     | 0.94 | 0.44 |
| 10.0           | 5.0     | 0.97 | 0.34 |
| 5.0            | 10.0    | 0.97 | 0.65 |
| 10.0           | 10.0    | 0.99 | 0.50 |

Supplement: S5 Fig — (A) Hep3B cells were treated with 0.1% DMSO (control) or various concentrations of corosolic acid for 24 h and cell viability was determined with an MTT assay. Results are presented as mean value ± SE. (***P < 0.001 compared with the DMSO treated group) (B) The migration activity of Hep3B cells was inhibited by corosolic acid in a dose-dependent manner. Results are presented as mean value ± SE. (**P < 0.01, ***P < 0.001 compared with the DMSO treated group) (C) Combinatorial effects of corosolic acid and sorafenib on Hep3B cell migration are displayed by CI value. (PDF) [file pone.0126725.s005.pdf]

## S6 Figure

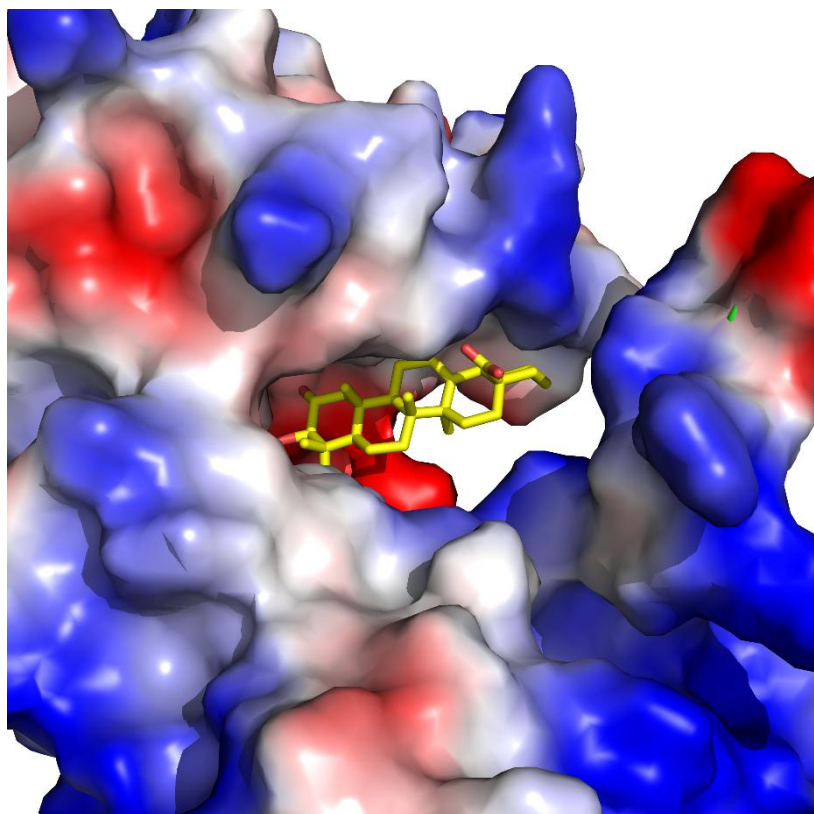

Supplement: S6 Fig — The surface charge distribution was displayed by PyMOL software. The negative charge, positive charge, and hydrophobic area were represented by red, blue, and white color, respectively. (PDF) [file pone.0126725.s006.pdf]

# S7 Figure

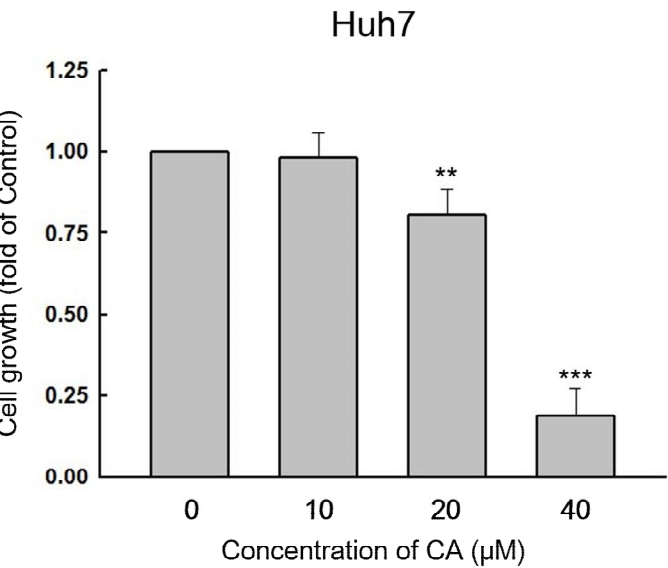

| Sorafenib (μM) | CA (μM) | (Fa) | (CI) |
|----------------|---------|------|------|
| 2.5            | 10.0    | 0.46 | 0.67 |
| 2.5            | 20.0    | 0.55 | 0.90 |
| 2.5            | 40.0    | 0.75 | 1.23 |
| 5.0            | 10.0    | 0.50 | 0.92 |
| 5.0            | 20.0    | 0.59 | 1.10 |
| 5.0            | 40.0    | 0.81 | 1.24 |
| 10.0           | 10.0    | 0.61 | 1.22 |
| 10.0           | 20.0    | 0.62 | 1.50 |
| 10.0           | 40.0    | 0.83 | 1.45 |

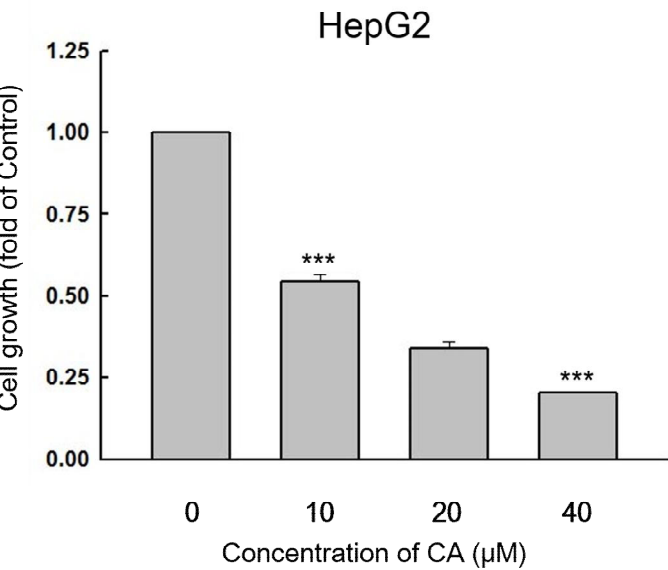

| Sorafenib (μM) | CA (μM) | (Fa) | (CI) |
|----------------|---------|------|------|
| 2.5            | 10.0    | 0.21 | 0.96 |
| 2.5            | 20.0    | 0.61 | 0.76 |
| 2.5            | 40.0    | 0.82 | 1.00 |
| 5.0            | 10.0    | 0.44 | 0.97 |
| 5.0            | 20.0    | 0.68 | 0.88 |
| 5.0            | 40.0    | 0.84 | 1.09 |
| 10.0           | 10.0    | 0.67 | 1.03 |
| 10.0           | 20.0    | 0.73 | 1.14 |
| 10.0           | 40.0    | 0.85 | 1.28 |

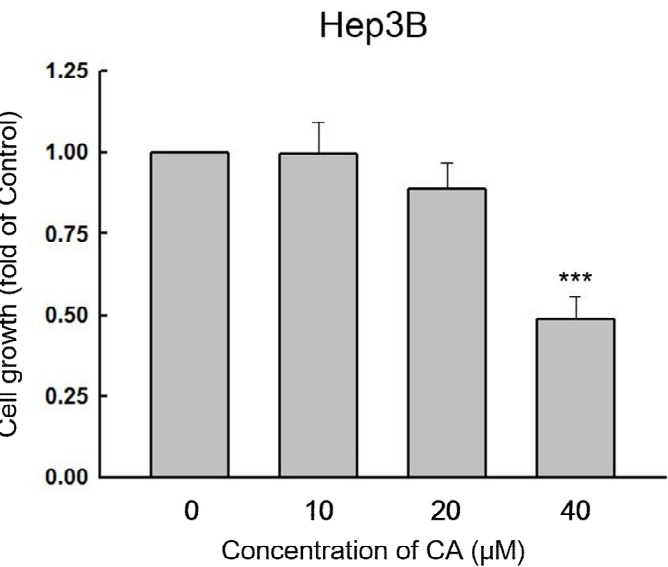

| Sorafenib (μM) | CA (μM) | (Fa) | (CI) |
|----------------|---------|------|------|
| 2.5            | 10.0    | 0.01 | 5.70 |
| 2.5            | 20.0    | 0.38 | 0.70 |
| 2.5            | 40.0    | 0.61 | 0.93 |
| 5.0            | 10.0    | 0.10 | 1.29 |
| 5.0            | 20.0    | 0.47 | 0.68 |
| 5.0            | 40.0    | 0.63 | 0.95 |
| 10.0           | 10.0    | 0.30 | 0.87 |
| 10.0           | 20.0    | 0.41 | 0.94 |
| 10.0           | 40.0    | 0.68 | 0.95 |

Supplement: S7 Fig — (A) Cells were treated with 0.1% DMSO (control) or varying concentrations of corosolic acid for 24 h, and the growth inhibition effect of corosolic acid was determined by SRB assay. Results are presented as mean value ± SE. (**P < 0.01, ***P < 0.001 compared with the DMSO treated group); combinatorial effects of corosolic acid and sorafenib on HCC cell growth are displayed on the right side of each chart. (PDF) [file pone.0126725.s007.pdf]

# S8 Figure

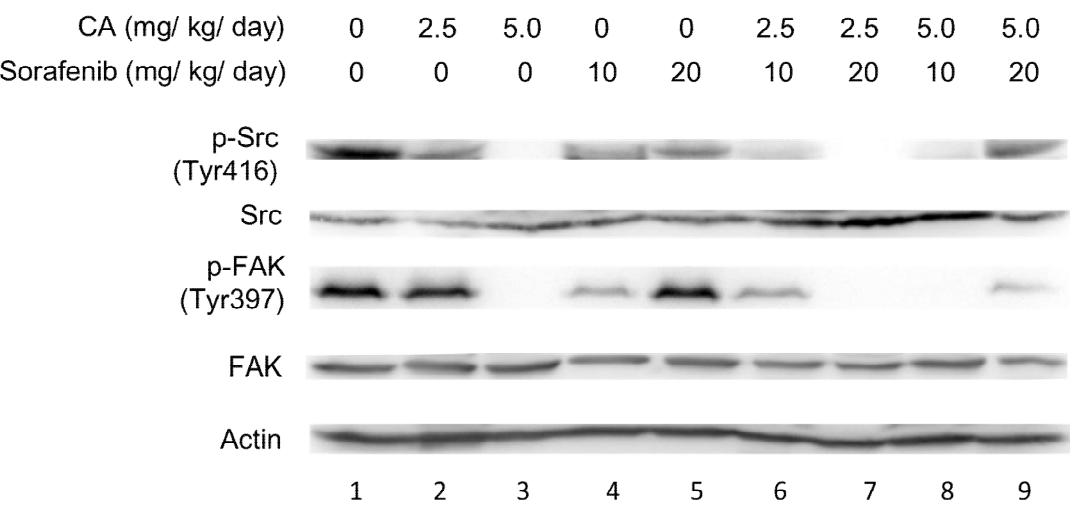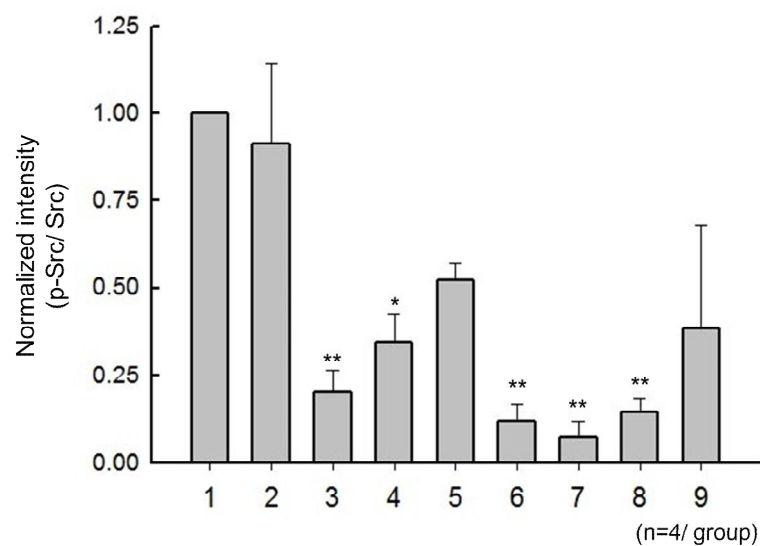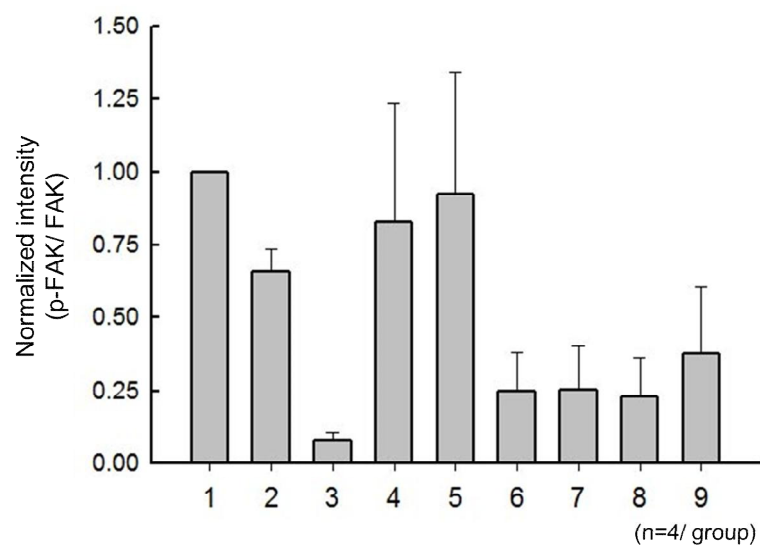

Supplement: S8 Fig — Xenograft tumors excised from mice were homogenized in RIPA buffer and analyzed by western blotting. (n = 4 for each group, *P < 0.05, **P < 0.01 compared with the DMSO treated control group) (PDF) [file pone.0126725.s008.pdf]

## S9 Figure

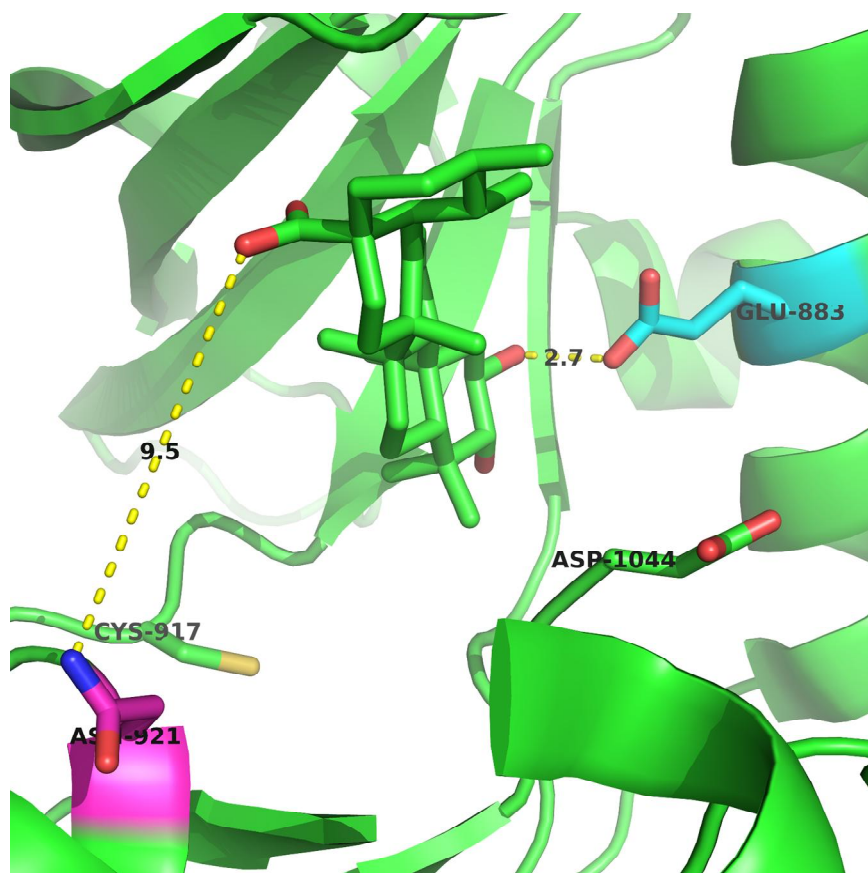

Supplement: S9 Fig — The Glu883 residue was represented by cyan color, and Asn921 residue was showed by pink color. The yellow dotted line means the distance between corosolic acid with these two residues. (PDF) [file pone.0126725.s009.pdf]
