# Supplementary material for: Corosolic Acid Inhibits Hepatocellular Carcinoma Cell Migration by Targeting the VEGFR2/Src/FAK Pathway
Source: PLoS One. 2015 May 15;10(5):e0126725. doi: 10.1371/journal.pone.0126725 (PMC4433267; doi:10.1371/journal.pone.0126725)

S3 Figure

Column type: RP-18 column
Mobile phase: 100% ddH_2O→100% MeOH
Injection volume: 20 μL
Elution rate: 1 mL/min
Detector: ELSD

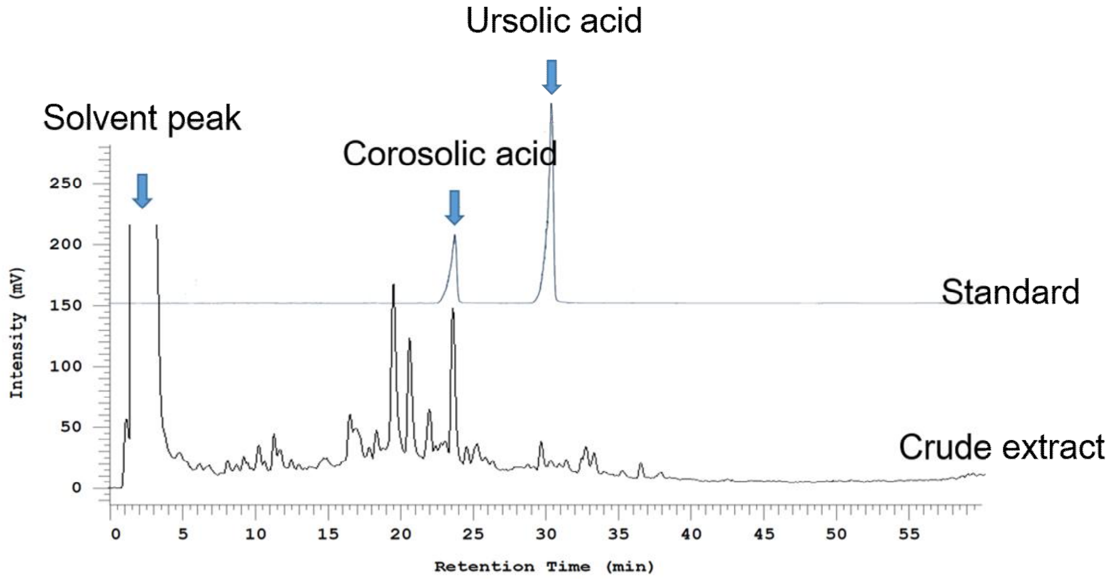

Supplement: S3 Fig — High-Performance liquid chromatography-diode array (HPLC-DAD)/ELSD chromatography was used to examine compounds in A. chinensis. The conditions for analysis are described in the methods section. (PDF) [file pone.0126725.s003.pdf]
